# Role of Adiponectin‐Notch pathway in cognitive dysfunction associated with depression and in the therapeutic effect of physical exercise

**DOI:** 10.1111/acel.13387

**Published:** 2021-05-30

**Authors:** Jingjing You, Linshan Sun, Jiangong Wang, Fengjiao Sun, Wentao Wang, Dan Wang, Xueli Fan, Dunjiang Liu, Zhicheng Xu, Changyun Qiu, Jinbo Chen, Haijing Yan, Bin Liu

**Affiliations:** ^1^ Institute for Metabolic & Neuropsychiatric Disorders Binzhou Medical University Hospital Shandong China; ^2^ Department of Neurology Binzhou Medical University Hospital Shandong China; ^3^ Department of Pharmacology College of Basic Medicine Binzhou Medical University Yantai China

**Keywords:** aging, behavior, chronic stimulation, cognition dysfunction, endocrinology, mouse models, neural stem cells, Notch

## Abstract

A substantial percentage of late‐life depression patients also have an cognitive impairment, which severely affects the life quality, while the co‐occurring mechanisms are still unclear. Physical exercise can ameliorate both depressive behaviors and cognitive dysfunction, but the molecular mechanisms underlying its beneficial effects remain elusive. In this study, we uncover a novel adipose tissue to hippocampus crosstalk mediated by Adiponectin‐Notch pathway, with an impact on hippocampal neurogenesis and cognitive function. Adiponectin, an adipocyte‐derived hormone, could activate Notch signaling in the hippocampus through upregulating ADAM10 and Notch1, two key molecules in the Notch signaling. Chronic stress inhibits the Adiponectin‐Notch pathway and induces impaired hippocampal neurogenesis and cognitive dysfunction, which can be rescued by AdipoRon and running. Inhibition Notch signaling by DAPT mimics the adverse effects of chronic stress on hippocampal neurogenesis and cognitive function. Adiponectin knockout mice display depressive‐like behaviors, associated with inhibited Notch signaling, impaired hippocampal neurogenesis and cognitive dysfunction. Physical exercise could activate Adiponectin‐Notch pathway, and improve hippocampal neurogenesis and cognitive function, while deleting adiponectin gene or inhibiting Notch signaling blocks its beneficial effects. Together, our data not only suggest that Adiponectin‐Notch pathway is involved in the pathogenesis of cognitive dysfunction associated with depression, but also contributes to the therapeutic effect of physical exercise. This work helps to decipher the etiology of cognitive impairment associated with depression and hence will provide a potential innovative therapeutic target for these patients.

## Graphical abstract

Adiponectin is secreted from adipose and can cross blood‐brain barrier and activates Notch siganling in the hippocampus. Chronic restraint stress decreases hippocampal neurogenesis and causes cognitive dysfunction through downregulation Adiponectin‐Notch signalling pathway, while physical exercise reverses the adverse effects of chronic restraint stress on hippocampal neurogenesis and cognitive function through upregulation of Adiponectin‐Notch signalling pathway.

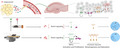

## 1 INTRODUCTION

Cognitive impairment is prevalent in late‐life depression and often persists even after remission of mood symptoms (Culpepper et al., 2017; Morimoto et al., 2015). The occurrence of depression in mild cognitive impairment (MCI) accelerates the progression to dementia (Rosenberg et al., 2013). However, little is known about the joint or individual mechanisms of co‐occurring depression and cognitive impairment. Previous studies have reported that physical exercise ameliorates depressive behaviors, enhances hippocampal neurogenesis, and improves hippocampal‐dependent learning and memory (Duzel et al., 2016; Yau et al., 2014). However, the mechanisms that mediate these effects of physical exercise remain largely unknown. Moreover, it is unclear which components of exercise programs are therapeutic.

Adiponectin (APN), a hormone secreted predominantly by adipocytes and playing critical roles in body energy homeostasis (Katsimpardi et al., 2020), decreases under chronic stress (Guo et al., 2017) and increases after exercise (Yau et al., 2014) in the circulation. Adiponectin could cross the blood‐brain barrier (Neumeier et al., 2007), exert neuroprotective and antidepressant properties through binding its receptors (Thundyil et al., 2012), AdipoR1, and AdipoR2, which are expressed in many brain regions. It has recently been found that adiponectin mimics many of the ameliorative effects of physical exercise on metabolism, hippocampal neurogenesis, depression and cognitive dysfunction (Greenhill, 2015; Liu et al., 2020; Nicolas et al., 2015; Yau et al., 2014). Adiponectin deficiency has been found to induce decreased hippocampal neurogenesis and cognitive dysfunction, and increase susceptibility to developing depressive behaviors under stress (Liu et al., 2012; Ng et al., 2016; Zhang et al., 2016). These studies indicate that adiponectin may be a candidate molecule involved in both depression and cognitive impairment induced by stress and may also be a therapeutic component of exercise programs.

There is a consensus that the hippocampus is essential in spatial learning and memory, as well as in the stress response and in the pathophysiology of mood‐related disorders (Cao et al., 2017; Lisman et al., 2017). Adult neurogenesis in the dentate gyrus of the hippocampus is of considerable importance for cognitive processes (Anacker, & Hen, 2017; Moreno‐Cugnon et al., 2019). Studies have shown that Notch signaling is essential in controlling a wide range of cellular processes during adult hippocampal neurogenesis (Ables et al., 2010; Breunig et al., 2007). Osmotin, a tobacco protein functionally similar to mammalian adiponectin, is shown to increase the protein levels of the α‐secretases, a disintergrin, and metalloprotease 10 and 17 (ADAM10 and ADAM17, key molecules in Notch signaling) in the hippocampus (Shah et al., 2017). However, whether Notch signaling is involved in adiponectin‐mediated hippocampal neurogenesis is unknown.

In this study, we showed that the modulation of adiponectin mediates the beneficial effects of physical exercise and adverse effects of chronic restraint stress on hippocampal neurogenesis and cognitive functions. Furthermore, we discovered a novel mechanism that adiponectin enhanced hippocampal neurogenesis through activating the Notch signaling.

## 2 RESULTS

### 2.1 Adiponectin deficiency leads to cognitive dysfunction in middle‐aged mice

**FIGURE 1:**
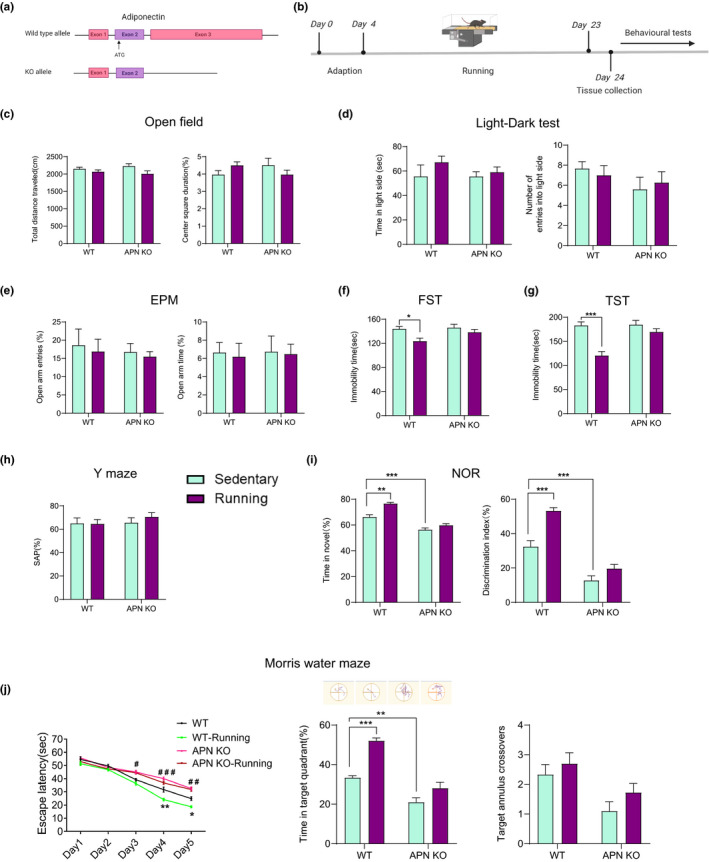
Adiponectin knockout induces cognitive dysfunction in middle‐aged mice and diminishes the beneficial effects of physical exercise on cognitive function. (a) A schematic of adiponectin knockout strategy. (b) The experimental timeline. (c) Open field test showing the effects of adiponectin knockout and running on locomotor activity and anxious level. Left, total distance travelled in 10 min, genotype, *F*_(1,36)_ = 0.01667, *p* = 0.8980; treatment, *F*_(1,36)_ = 5.011, *p* = 0.0315; genotype × treatment, *F*_(1,36)_ = 1.075, *p* = 0.3066; Right, percentage of time spent in the center zone, genotype, *F*_(1,36)_ = 0.0002784, *p* = 0.9868. Treatment, *F*_(1,36)_ = 2.477e‐6, *p* = 0.9988; genotype × treatment, *F*_(1,36)_ = 3.617, *p* = 0.0652. (d) Light‐dark test showing the effects of adiponectin knockout and running on anxious level. Left, time spent in the light compartment in 5 min, genotype, *F*_(1,36)_ = 0.5116, *p* = 0.4791; treatment, *F*_(1,36)_ = 1.747, *p* = 0.1946; genotype × treatment, *F*_(1,36)_ = 0.4954, *p* = 0.4861; Right, the number of entries into the light compartment, genotype, *F*_(1,36)_ = 1.856, *p* = 0.1815; treatment, *F*_(1,36)_ = 8.734e‐6, *p* = 0.9977; genotype × treatment, *F*_(1,36)_ = 0.4266, *p* = 0.5178. (e) the elevated plus maze (EPM) test showing the effects of adiponectin knockout and running on anxious level. Left, percentage of entries into the open arms, genotype, *F*_(1,36)_ = 0.3064, *p* = 0.5833; treatment, *F*_(1,36)_ = 0.2544, *p* = 0.6171; genotype × treatment, *F*_(1,36)_ = 0.006721, *p* = 0.9351; Right, percentage of the time spent on the open arms, genotype, *F*_(1,36)_ = 0.01916, *p* = 0.8907; treatment, *F*_(1,36)_ = 0.06779, *p* = 0.7961; genotype × treatment, *F*_(1,36)_ = 0.004680, *p* = 0.9458. (f) The forced swim test (FST) showing the effects of adiponectin knockout and running on depression level genotype, *F*_(1,36)_ = 3.080, *p* = 0.0878; treatment, *F*_(1,36)_ = 8.569, *p* = 0.0059; genotype × treatment, *F*_(1,36)_ = 1.751, *p* = 0.1941. (g) Tail suspension test (TST) showing the effects of adiponectin knockout and running on depression level genotype, *F*_(1,36)_ = 10.75, *p* = 0.0023; treatment, *F*_(1,36)_ = 25.27, *p* < 0.0001; genotype × treatment, *F*_(1,36)_ = 9.520, *p* = 0.0039. (h) Y‐maze test showing the effects of adiponectin knockout and running on the percentage of correct spontaneous alternation (SAP), genotype, *F*_(1,36)_ = 0.6703, *p* = 0.4183; treatment, *F*_(1,36)_ = 0.3169, *p* = 0.5769; genotype × treatment, *F*_(1,36)_ = 0.4390, *p* = 0.5118. (i) Novel objective recognition (NOR) test showing the effects of adiponectin knockout and running on the recognition memory of the mice. Left, percentage of time spent on the novel object, genotype, *F*_(1,36)_ = 104.3, *p* < 0.001; treatment, *F*_(1,36)_ = 28.25, *p* < 0.001; genotype × treatment, *F*_(1,36)_ = 7.159, *p* = 0.0112; Right, the discrimination index, *F*_(1,36)_ = 104.3, *p* < 0.001; treatment, *F*_(1,36)_ = 28.25, *p* < 0.001; genotype × treatment, *F*_(1,36)_ = 7.159, *p* = 0.0112. WT sedentary, *n* = 9; WT Running, *n* = 10; APN‐KO sedentary, *n* = 10; APN‐KO Running, *n* = 11 in c–i. Mice of all groups were 12‐month‐old. *p < 0.05, **p < 0.01, ***p < 0.001. (j) Morris water maze test showing the escape latency on training days (left, subgroups, *F*_(3,36)_ = 42.52, *p* < 0.001; time, *F*_(4,144)_ = 239.6, *p* < 0.001; subgroups × time, *F*_(12,144)_ = 4.962, *p* < 0.001), illustrative examples of travel pathways for each group (upper middle) and percentage of time spent in target quadrant (lower middle, genotype, *F*_(1,36)_ = 36.35, *p* < 0.001; treatment, *F*_(1,36)_ = 72.67, *p* < 0.001; genotype × treatment, *F*_(1,36)_ = 7.330, *p* = 0.0103), the times crossing the platform on testing day (right, genotype, *F*_(1,36)_ = 2.258, *p* = 0.1417; treatment, *F*_(1,36)_ = 11.12, *p* = 0.0020; genotype × treatment, *F*_(1,36)_ = 0.1552, *p* = 0.6959). WT sedentary, *n* = 9; WT Running, *n *= 10; APN‐KO sedentary, *n* = 10; APN‐KO Running, *n* = 10. Mice of all groups were 12‐month‐old. *p < 0.05, **p < 0.01 for WT sedentary versus WT running and ^#^ *p* < 0.05, ^##^ *p* < 0.01, ^###^ *p* < 0.001 for WT sedentary versus APN‐KO sedentary in the left; **p < 0.01, ***p < 0.001 in the middle

**FIGURE 2:**
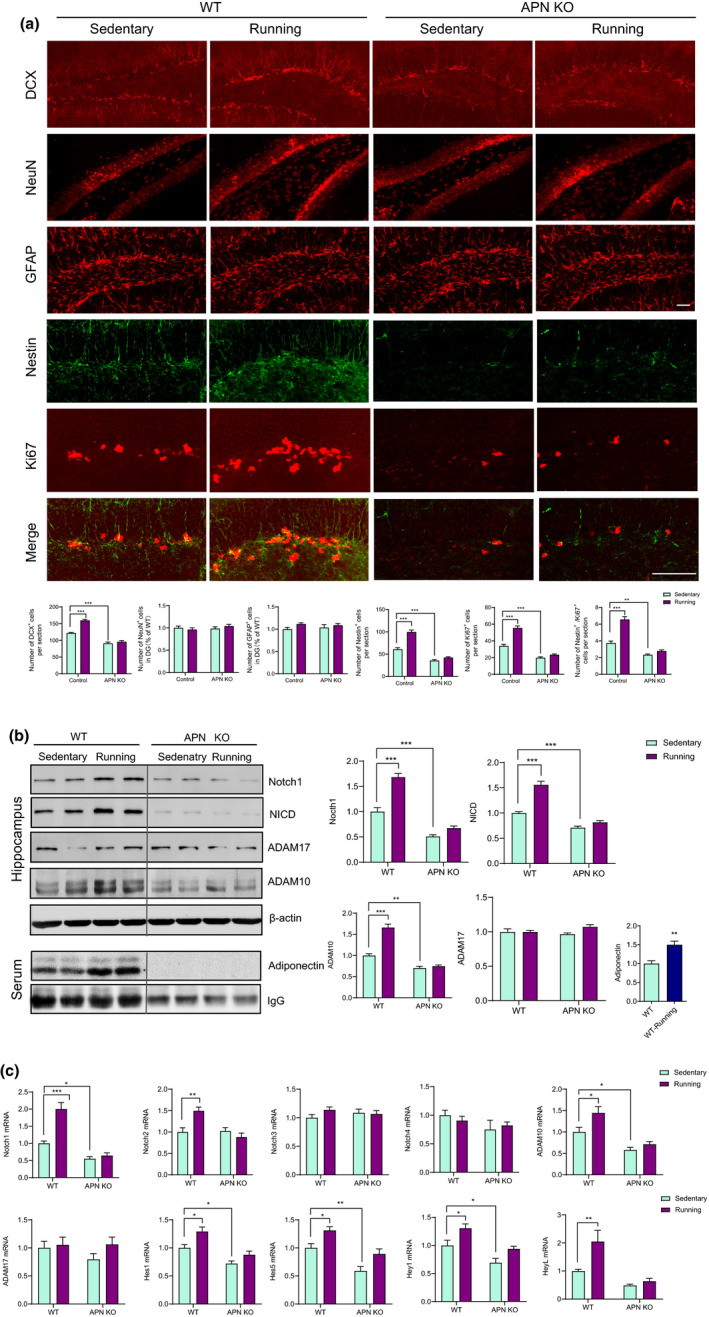
Adiponectin knockout induces impaired hippocampal neurogenesis and decreased hippocampal Notch signaling in middle‐aged mice and diminishes the beneficial effects of physical exercise on hippocampal neurogenesis and Notch signaling. (a) Upper, representative images of immunohistochemical staining of DCX, NeuN, GFAP, Nestin and Ki67 in DG; Lower, statistical analysis of the histologic data, for DCX, genotype, *F*_(1,22)_ = 41.49, *p* < 0.001; treatment, *F*_(1,22)_ = 205.6, *p* < 0.001; genotype × treatment, *F*_(1,22)_ = 24.75, *p* = 0.001; for NeuN, genotype, *F*_(1,22)_ = 0.05052, *p* = 0.8242; treatment, *F*_(1,22)_ = 0.6114, *p* = 0.4426; genotype × treatment, *F*_(1,22)_ = 1.394, *p* = 0.2503; for GFAP, genotype, *F*_(1,22)_ = 3.467, *p* = 0.0760; treatment, *F*_(1,22)_ = 0.009812, *p* = 0.9220; genotype × treatment, *F*_(1,22)_ = 0.4891, *p* = 0.4916; for Nestin, genotype, *F*_(1,22)_ = 48.26, *p* < 0.0001; treatment, *F*_(1,22)_ = 160.6, *p* < 0.0001; genotype × treatment, *F*_(1,22)_ = 24.31, *p* < 0.0001; for Ki67, genotype, *F*_(1,22)_ = 47.67, *p* < 0.0001; treatment, *F*_(1,22)_ = 160.1, *p* < 0.0001; genotype × treatment, *F*_(1,22)_ = 24.19, *p* < 0.0001; for Nestin+/Ki67+, genotype, *F*_(1,22)_ = 49.77, *p* < 0.0001; treatment, *F*_(1,22)_ = 126.2, *p* <0.0001; genotype × treatment, *F*_(1,22)_ = 25.95, *p* < 0.0001. WT sedentary, *n* = 6; WT Running, *n* = 7; APN‐KO sedentary, *n* = 6; APN‐KO Running, *n* = 7. Mice of all groups were 12‐month‐old. **p < 0.01, ***p < 0.001. Data are presented as means ± SEM. (b) Representative immunoblots and quantification of hippocampal (including Notch1, NICD, ADAM10, ADAM17) and serum protein levels (adiponectin). (c) qRT‐PCR analysis of gene expression for components of Notch signaling pathway in hippocampus, including Notch1‐4, ADAM10, ADM17, Hes1, Hes5, Hey1 and HeyL. WT sedentary, *n* = 6; WT Running, *n* = 6; APN‐KO sedentary, *n* = 7; APN‐KO Running, *n* = 7. *p < 0.05, **p < 0.01, ***p < 0.001. Mice of all groups were 12‐month‐old. Data are presented as means ± SEM. Scale bar: f, 50 μm

The mouse adiponectin gene includes three exons that encode a 247 amino acid protein. Adiponectin consists of an N‐terminal signal sequence, a hypervariable region, a collagenous domain, and a C1q‐like globular domain. The globular domain, encoded by exon 3, facilitates the binding of adiponectin to its receptors (Thundyil et al., 2012) and exerts similar biologic activity as full‐length adiponectin after release by the enzyme leukocyte elastase (Fruebis et al., 2001; Waki et al., 2005). In our study, the exon 3 of adiponectin gene, containing 521bp coding sequence for globular domain and part of collagenous domain, was knocked out using CRISPR/Cas9 technology (Figure 1a). As shown in Figure 2b, we didn't detect adiponectin in the serum of the knockout (APN‐KO) mice.

Previous studies have shown that adiponectin deficiency in middle‐aged mice leads to spatial learning and memory impairments, in which the used APN‐KO mice lines are different from ours (Bloemer et al., 2019; Ng et al., 2016). To determine whether cognitive deficits are also present in our middle‐aged APN‐KO mice, we performed an open field test, light‐dark test, elevated plus maze (EPM), forced swim test (FST), Tail suspension test (TST), Y‐maze, novel object recognition (NOR) and Morris water maze tests with 12‐month‐old APN‐KO mice. The open field test, light‐dark test, and EPM were used to evaluate the anxiety level. In the open field test, there was no significant difference in the total distance traveled (Figure 1c, left) or time spent in the center area between APN‐KO mice and wild‐type (WT) littermate mice (Figure 1c, right). Adiponectin deficiency did not significantly affect the total time spent in the light compartment (Figure 1d, left) and the number of entries into the light compartments (Figure 1d, right). EPM also showed that adiponectin deficiency didn't affect the percentage of open arm entries (Figure 1e, left) and the percentage of open arm time (Figure 1e, right). Collectively, these data suggested that adiponectin deficiency didn't affect the anxiety level in middle‐aged mice (12‐month‐old). The FST and TST results showing that adiponectin deficiency also didn't affect the depressive level in middle‐aged mice (Figure 1f,g). The results of the Y‐maze test showed that the total percentage of correct spontaneous alterations percentage (SAP) did not differ significantly between APN‐KO mice and WT littermates (Figure 1h). In the NOR test, APN‐KO mice spent less time on novel object investigation than WT littermates (Figure 1i). The results of the Morris water maze test showed that the escape latency of APN‐KO mice was significantly increased compared with that of WT littermates on day 3–day 5 (Figure 1j, left) and that APN‐KO mice spent less time in the target quadrant than WT littermates on the test day (Figure 1j, lower middle). The numbers of target annulus crossovers revealed a decreasing but nonsignificant trend for APN‐KO mice compared with WT littermates (Figure 1j, right). Consistent with previous reports, our 12‐month‐old APN‐KO mice also displayed cognitive dysfunction, as evaluated by the NOR and Morris water maze tests.

### 2.2 Adiponectin deficiency leads to attenuated hippocampal neurogenesis in middle‐aged mice

Studies have indicated that hippocampal neurogenesis plays a key role in learning and memory (Alam et al., 2018). Thus, we subsequently investigated by Ki67 (cell proliferation marker), Nestin (marker for neural stem cells), Doublecortin (DCX, marker for newly born young neurons), NeuN (marker for mature neurons), and GFAP (marker for mature Astrocytes) staining whether the behavioral alterations were correlated with corresponding changes in hippocampal neurogenesis. As shown in Figure 2a, we observed decreased numbers of DCX^+^, Ki67^+^, Nestin^+^, and Nestin^+^/Ki67^+^ cells in aged APN‐KO mice compared with WT littermates, while there were no significant differences in the numbers of mature neurons and astrocytes in DG. These results indicated that adiponectin deficiency was associated with attenuated hippocampal neurogenesis which may contribute to the cognitive impairment in middle‐aged (12‐month‐old) APN‐KO mice.

### 2.3 Adiponectin deficiency diminishes the beneficial effects of physical exercise on cognitive performance and hippocampal neurogenesis

Studies have indicated that physical exercise can increase adult hippocampal neurogenesis by adiponectin‐dependent pathway (Yau et al., 2014) and improve cognitive functions in humans (Kwok et al., 2019), indicating adiponectin may be involved in the physical exercise‐induced improvement of cognition. To clarify the role of adiponectin in cognitive improvement, we investigated whether physical exercise could ameliorate the functional impairment of spatial learning and memory in 12‐month‐old APN‐KO mice. Running significantly increased the time spent by WT littermate mice but not APN‐KO mice (Figure 1i) on novel object investigation. Similar results were also observed in the Morris water maze test. Running significantly decreased the escape latency of WT littermate mice on days 4–5 (Figure 1j, left) and increased the time spent by these mice in the target quadrant (Figure 1j, lower middle), but these effects were markedly diminished by adiponectin knockout (Figure 1j). Running did not influence the behavioral performance of either WT or APN‐KO mice in the Y‐maze test (Figure 1h). Running exerted antidepressant effect on WT littermate mice evaluated by FST (Figure 1f) and TST (Figure 1g), while this effect was diminished by adiponectin knockout. The open field (Figure 1c), light‐dark test (Figure 1d), and EPM (Figure 1e) results showed that running did not influence the anxiety level of either WT or APN‐KO mice. Taken together, these results indicated that adiponectin was required for running‐elicited improvement in both cognitive performance and antidepressant effect in middle‐aged mice. To determine whether adiponectin also plays a role in physical exercise‐induced hippocampal neurogenesis in middle‐aged mice as in young mice (Yau et al., 2014), immunofluorescence staining for Ki67 and DCX was performed in hippocampal slices. Running significantly increased the number of Ki67‐, Nestin‐, and DCX‐positive cells in WT mice, but not in APN‐KO mice (Figure 2a). This pattern suggested that adiponectin was required for running‐induced hippocampal neurogenesis in middle‐aged mice. Collectively, these results suggested that adiponectin was required for the beneficial effects of physical exercise on neurogenesis and cognition in middle‐aged mice.

### 2.4 Decreased serum adiponectin level associated with impairments in hippocampal neurogenesis and cognitive function in aged mice

**FIGURE 3:**
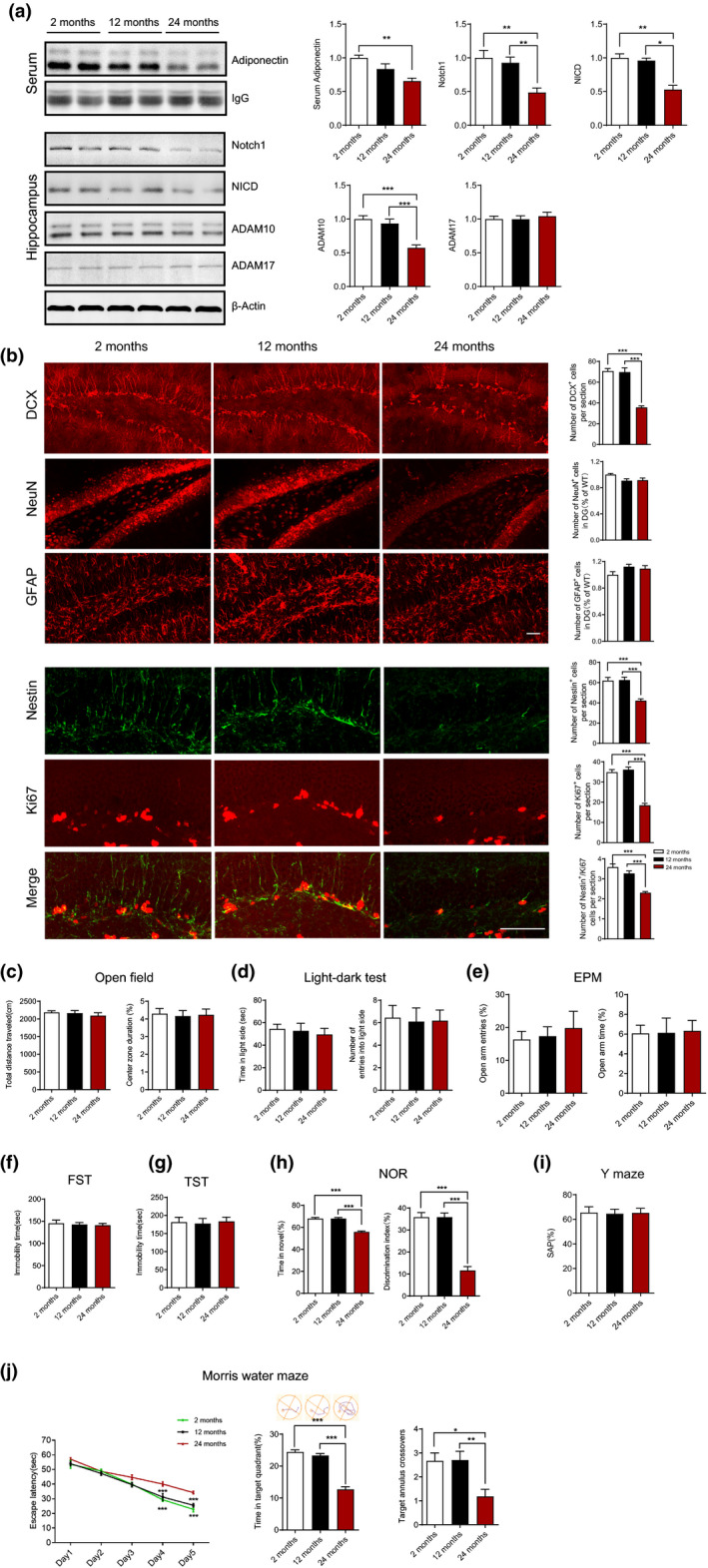
Adiponectin decreases in aged mice displaying decreased hippocampal Notch signaling, impaired neurogenesis, and cognitive dysfunction. (a) Representative immunoblots and quantification of hippocampal (including Notch1, NICD, ADAM10, ADAM17) and serum (adiponectin) protein levels. 2 months, *n* = 6; 12 months, *n* = 7; 24 months, *n* = 7. *p < 0.05, **p < 0.01, ***p < 0.001. (b) Representative images of immunohistochemical staining of DCX, NeuN, GFAP, Nestin and Ki67 in DG and statistical analysis of the histologic data. 2 months, *n* = 6; 12 months, *n* = 7; 24 months, *n* = 6. ***p < 0.001. (c) Open field test showing the locomotor activity and anxious level. Left, total distance travelled in 10 min, Kruskal–Wallis test, *p* = 0.6239; Right, percentage of time spent in the center zone, *F*_(2,27)_ = 0.04517, *p* = 9559. (d) Light‐dark test showing the anxious level. Left, time spent in the light compartment in 5 min, *F*_(2,27)_ = 0.1908, *p* = 0.8274; Right, the number of entries into the light compartment, *F*_(1,36)_ = 0.02592, *p* = 0.9744. (e) the elevated plus maze (EPM) test showing the anxious level. Left, percentage of entries into the open arms, Kruskal–Wallis test, *p* = 0.9867; Right, percentage of the time spent on the open arms, Kruskal–Wallis test, *p* = 0.8011. (f) The forced swim test (FST) showing the depression level. *F*_(2,27)_ = 0.1758, *p* = 0.8397. (g) Tail suspension test (TST) showing the depression level. *F*_(2,27)_ = 0.05421, *p* = 0.9473. (h) Novel objective recognition (NOR) test showing the recognition memory of the mice. Left, percentage of time spent on the novel object, genotype, *F*_(2,27)_ = 54.99, *p* < 0.001; Right, the discrimination index, *F*_(2,27)_ = 54.99, *p* < 0.001. (i) Y‐maze test showing the percentage of correct spontaneous alternation (SAP), *F*_(2,27)_ = 0.1417, *p* = 0.9859. 2 months, *n* = 9; 12 months, *n* = 10; 24 months, *n* = 11 in c–i. ***p < 0.001. (j) Morris water maze test showing the escape latency on training days (left, subgroups, *F*_(2,27)_ = 24.86, *p* < 0.001; time, *F*_(4,108)_ = 144.4, *p* < 0.001; subgroups × time, *F*_(8,108)_ = 2.614, *p* = 0.0118), illustrative examples of travel pathways for each group (upper middle) and percentage of time spent in target quadrant (lower middle, *F*_(2,27)_ = 82.28, *p* < 0.001), the times crossing the platform on testing day (right, *F*_(1,36)_ = 7.141, *p* = 0.0032). 2 months, *n *= 9; 12 months, *n* = 10; 24 months, *n* = 11. *p < 0.05, **p < 0.01, ***p < 0.001. Data are presented as means ± SEM. Scale bar: f, 50 μm

As cognitive impairment and dementia are age‐related, we next determine whether adiponectin level was correlated with cognitive function in aged mice. The serum adiponectin level was significantly decreased in aged mice (24 months), but not in middle‐aged mice (12 months; Figure 3a). Consistent with the above results, aged mice, decreased serum adiponectin level, displayed inhibited hippocampal neurogenesis (Figure 3b) and cognitive dysfunction (Figure 3h–j). Decreased adiponectin level with aging didn't influence the anxiety level (Figure 3c–e) and depressive level (Figure 3f and g). Collectively, these data showed that adiponectin level was correlated with hippocampal neurogenesis and cognitive function, suggesting decreased adiponectin level may contribute to the age‐related cognitive dysfunction.

### 2.5 Adiponectin is required for physical exercise‐induced activation of the Notch signaling pathway in the hippocampus

We next explored the molecular mechanisms underlying the decreased neurogenesis in middle‐aged APN‐KO mice and adiponectin‐induced neurogenesis after running. Notch signaling plays an important role in adult hippocampal neurogenesis (Ables et al., 2010; Breunig et al., 2007), but there is no information on whether adiponectin can regulate Notch signaling. A previous report has shown that Osmotin, plant homolog of adiponectin, can increase the expression of ADAM10 and ADAM17, two rate‐limiting S2 enzymes for Notch cleavage, in the hippocampus of APP/PS1 mice (Shah et al., 2017), providing an indirect clue to the hypothesis that adiponectin could regulate Notch signaling. We thus examined the expression levels of several key molecules in the Notch pathway in the hippocampus, including four Notch receptors (Notch1‐4), the activated Notch intracellular domain (NICD), ADAM10, ADAM17, and downstream genes of Notch signaling (Hes1, Hes5, Hey1, and Heyl). The Western blot results showed that the protein levels of full‐length Notch1, NICD, and ADAM10 in WT mice were higher than those in APN‐KO mice (Figure 2b). Additionally, running significantly increased the levels of full‐length Notch1, NICD, and ADAM10 in WT mice but not in APN‐KO mice (Figure 2b). However, the level of the other S2 enzyme, ADAM17, was similar in WT and APN‐KO mice and was not significantly affected by running in either mouse strain (Figure 2b). Moreover, the serum level of adiponectin increased after running (Figure 2b). Similarly, the qPCR results showed that the mRNA levels of Notch1, ADAM10, and downstream genes of Notch signaling (Hes1, Hes5, Hey1, and Heyl) decreased in APN‐KO mice (Figure 2c). Running significantly increased the levels of Notch1, Notch2, ADAM10, Hes1, Hes5, Hey1, and Heyl in WT mice but not in APN‐KO mice (Figure 2c). The mRNA level of ADAM17 did not differ significantly between WT and APN‐KO mice and was not affected by running in either mouse strain (Figure 2c). Taken together, these results showed that adiponectin deficiency was associated with decreased Notch signaling, and running‐induced activation of Notch signaling in the hippocampus, associated with increased serum adiponectin level, was compromised in APN‐KO mice, indicating that adiponectin was a novel regulator of Notch signaling and involved in the effect of physical exercise on Notch signaling.

### 2.6 AdipoRon activates Notch signaling in the hippocampus

**FIGURE 4:**
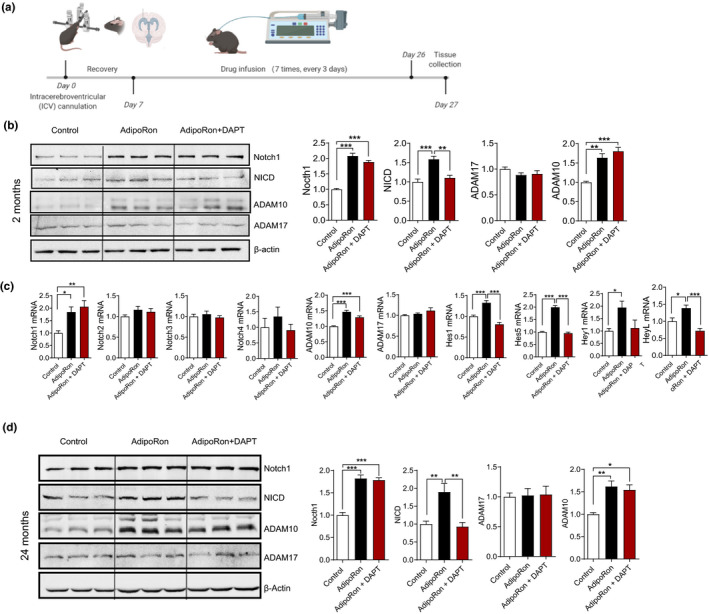
ICV injection of AdipoRon activates the Notch signaling pathway in the hippocampus in both young mice and aged mice. (a) The experimental timeline. (b) Representative immunoblots and quantification of hippocampal protein levels after ICV injection of AdipoRon in 2‐month‐old mice, including Notch1, NICD, ADAM10, and ADAM17. (c) qRT‐PCR analysis of gene expression for components of a Notch signaling pathway in hippocampus after ICV injection of AdipoRon in 2‐month‐old mice, including Notch1‐4, ADAM10, ADM17, Hes1, Hes5, Hey1, and HeyL. Control, *n* = 6; AdipoRon, *n* = 6; AdipoRon + DAPT, *n* = 6 in b and c. (d) Representative immunoblots and quantification of hippocampal protein levels after ICV injection of AdipoRon in 24‐month‐old mice, including Notch1, NICD, ADAM10, and ADAM17. Control, *n* = 6; AdipoRon, *n* = 6; AdipoRon + DAPT, *n* = 6. *p < 0.05, **p < 0.01, ***p < 0.001. Data are presented as means ± SEM

To clarify the effect of adiponectin on Notch signaling in the hippocampus, we performed intraventricular cannulation and intracerebroventricular (ICV) injection of AdipoRon (1 µl/1 mM), an adiponectin receptor agonist (Okada‐Iwabu et al., 2013), in 2‐month or 24‐month‐old C57BL/6J mice (Figure 4a). The Western blot results showed that the levels of full‐length Notch1, NICD, and ADAM10 were significantly increased by AdipoRon, and that DAPT (1 µl/40 µM), inhibiting the release of NICD from Notch receptors by targeting γ‐secretase (Dai et al., 2019), suppressed the AdipoRon‐induced increase of NICD without influencing the increases in Notch1 and ADAM10 expression in young mice (Figure 4b). The qPCR results showed that the mRNA levels of Notch1, ADAM10, Hes1, Hes5, Hey1, and Heyl were increased by AdipoRon and that DAPT blocked these increases in the mRNA levels of Hes1, Hes5, Hey1, and Heyl (Figure 4c). ADAM17 expression was not affected by AdipoRon (Figure 4b,c). Similar results were observed in aged (24 months) mice (Figure 4d). These results suggest that AdipoRon may increase NICD release through upregulation of Notch1 and ADAM10, subsequently activating the expression of Notch signaling target genes.

### 2.7 The Notch signaling mediates the adiponectin‐dependent beneficial effects of physical exercise on hippocampal neurogenesis and cognitive function

**FIGURE 5:**
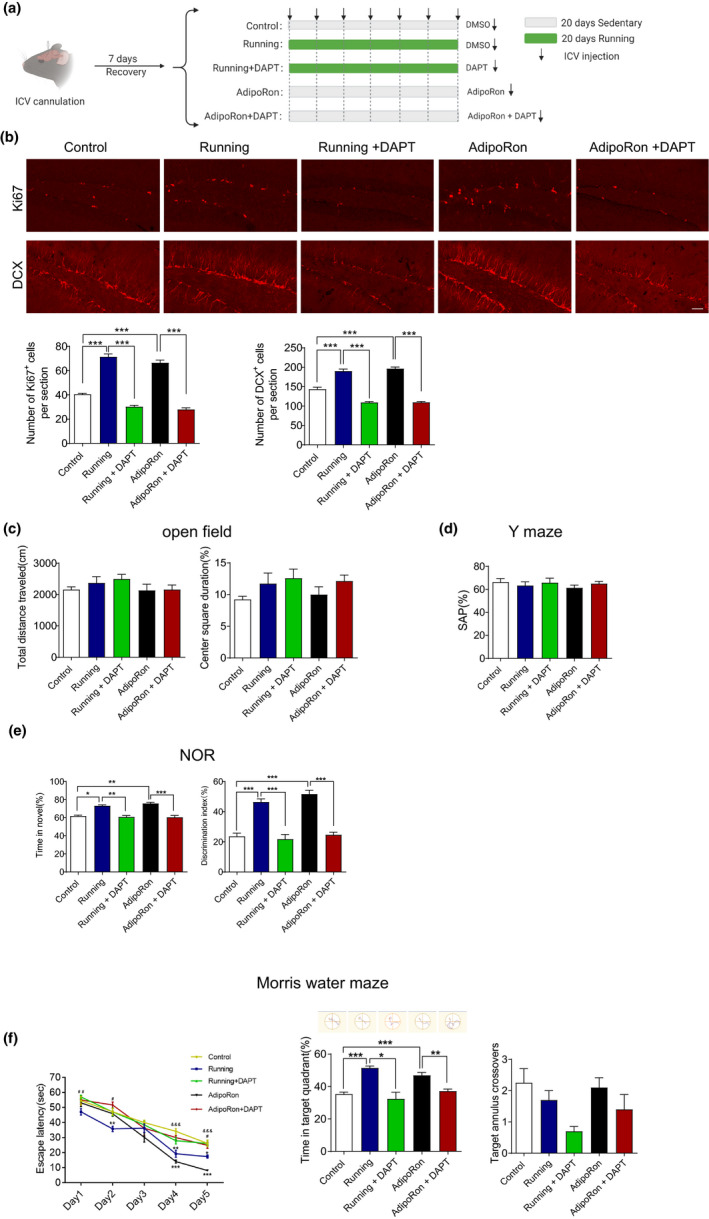
Notch signaling pathway is required for the beneficial effects of physical exercise and AdipoRon on hippocampal neurogenesis and cognitive function. (a) The experimental protocol and groups used. (b) representative image and quantification of immunohistochemical staining of Ki67 and DCX in DG. For, Ki67, *F*_(4,27)_ = 151.7, *p* < 0.001; For DCX, *F*_(4,27)_ = 11, *p* < 0.001. Control, *n* = 6; Running, *n* = 6; Running + DAPT, *n* = 7; AdipoRon, *n* = 7; AdipoRon + DAPT, *n* = 6. Mice of all groups were 2‐month‐old. *p < 0.05, **p < 0.01, ***p < 0.001. (c) Open field test showing the locomotor activity and anxious level after various treatments. Left, total distance travelled in 10 min, *F*_(4,43)_ = 0.9855, *p* = 0.4256; Right, percentage of time spent in the center zone, Kruskal–Wallis test, *p* = 0.1463. (d) Y‐maze test showing the percentage of correct spontaneous alternation after various treatments, *F*_(4,43)_ = 0.4493, *p* = 0.7723. (e) Novel objective recognition test showing the exploring time on new object after various treatments. Kruskal–Wallis test, *p* < 0.001. Control, *n* = 8; Running, *n* = 10; Running + DAPT, *n* = 10; AdipoRon, *n* = 10; AdipoRon + DAPT, *n* = 10 in c, d, and e. Mice of all groups were 2‐month‐old. *p < 0.05, **p < 0.01, *** *p* <0.001 in e. (f) Morris water maze test showing the escape latency on training days (left, subgroups, *F*_(4,43)_ = 15.74, *p* < 0.001; time, *F*_(4,172)_ = 202.5, *p* < 0.001; subgroups × time, *F*_(16,172)_ = 3.673, *p* = 0.001), illustrative examples of travel pathways for each group (upper middle) and percentage of time spent in target quadrant (lower middle, *F*_(4,43)_ = 13.22, *p* < 0.001), the times crossing the platform on testing day (right, Kruskal–Wallis test, *p* = 0.0772). Control, *n* = 8; Running, *n* = 10; Running + DAPT, *n* = 10; AdipoRon, *n* = 10; AdipoRon + DAPT, *n* = 10. Mice of all groups were 2‐month‐old. In the left, *p < 0.05, **p < 0.01, ***p < 0.001 for Running versus Control and AdipoRon versus Control; ^#^ *p* < 0.05, ^##^ *p* < 0.01 for Running versus Running + DAPT; ^&&&^ *p* < 0.001 for AdipoRon versus AdipoRon + DAPT. In the middle, *p < 0.05, **p < 0.01, ***p < 0.001. Data are presented as means ± SEM. Scale bar: b, 50 μm

We next determined whether the Notch signaling was involved in adiponectin‐dependent improvements in hippocampal neurogenesis and cognition induced by running. As shown in Figure 5b, both running and AdipoRon treatment increased the numbers of Ki67‐ and DCX‐positive cells, and DAPT blocked the enhanced hippocampal neurogenesis induced by running or AdipoRon treatment in 2‐month‐old mice. These results suggested that Notch signaling mediated the effects of AdipoRon and running on hippocampal neurogenesis. In the NOR test, both running and AdipoRon treatment were found to significantly increase the time spent on novel object investigation, and this effect was blocked by DAPT in 2‐month‐old mice (Figure 5e). In the Morris water maze test, both running and AdipoRon treatment significantly decreased the escape time during the training period (Figure 5f, left) and increased the time spent in the target quadrant on the test day (Figure 5f, lower middle) in 2‐month‐old mice. Similarly, DAPT blocked the effects of running and AdipoRon treatment (Figure 5f, left and middle). However, the number of target annulus crossovers did not differ significantly (Figure 5f, right). Running and AdipoRon did not influence the behavioral performance in the open field (Figure 5c) or Y‐maze test (Figure 5d) in 2‐month‐old mice. These behavioral tests indicated that Notch signaling was required for improvement of cognitive function induced by AdipoRon or running. Collectively, our results suggested that Adiponectin‐Notch pathway mediated the beneficial effects of physical exercise on hippocampal neurogenesis and cognitive function.

### 2.8 Adiponectin‐Notch pathway was involved in both cognitive dysfunction associated with depression and the therapeutic effect of physical exercise

Given the importance of Adiponectin‐Notch pathway in hippocampal neurogenesis and cognitive function under basal conditions and during physical exercise, we next assessed whether Adiponectin‐Notch pathway was also involved in cognitive dysfunction associated with depression induced by chronic stress in middle‐aged mice.

**FIGURE 6:**
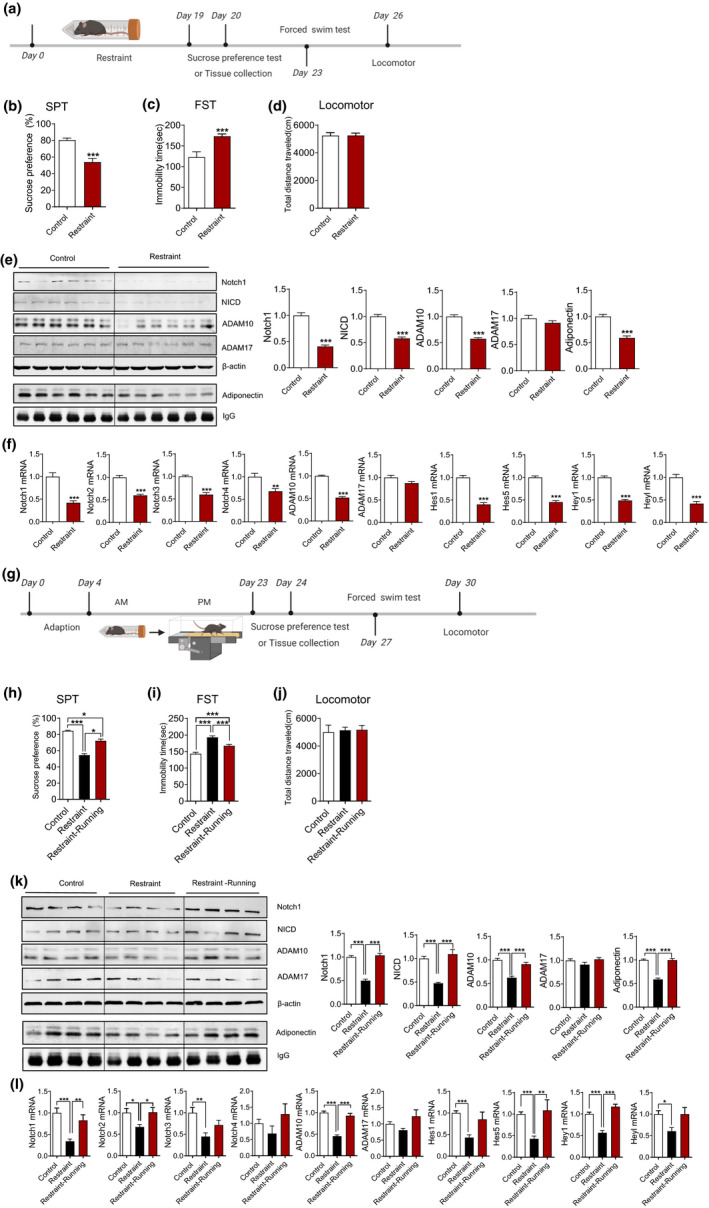
Physical exercise reverses the decreased Notch signaling pathway in the hippocampus induced by chronic restraint stress. (a) The experimental timeline. (b) Sucrose preference test. (c) Forced swim test. (d) Locomotor activity. Total distance traveled in 30 min. Control, *n* = 8; Restraint, *n* = 12 in b, c, and d. Mice of all groups were 2‐month‐old. ***p < 0.001 in b and c. (e) Representative immunoblots and quantification of hippocampal (including Notch1, NICD, ADAM10, ADAM17) and serum protein levels (adiponectin). (f) qRT‐PCR analysis of gene expression for components of a Notch signaling pathway in hippocampus after restraint, including Notch1‐4, ADAM10, ADM17, Hes1, Hes5, Hey1, and HeyL Control, *n* = 8; Restraint, *n* = 9 in e and f. Mice of all groups were 2‐month‐old. ***p < 0.001 in e and f. (g) The experimental timeline. (h) Sucrose preference test. (i) Forced swim test. (j) Locomotor activity. Total distance traveled in 30 min. Control, *n* = 10; Restraint, *n* = 12; Restraint + Running, *n* = 12 in b, c, and d. Mice of all groups were 2‐month‐old. *p < 0.05, ***p < 0.001 in h and i. (k) Representative immunoblots and quantification of hippocampal (including Notch1, NICD, ADAM10, ADAM17) and serum protein levels (adiponectin). (l) qRT‐PCR analysis of gene expression for components of a Notch signaling pathway in hippocampus, including Notch1‐4, ADAM10, ADM17, Hes1, Hes5, Hey1, and HeyL Control, *n* = 10; Restraint, *n* = 10; Restraint + Running, *n* = 10 in e and f. Mice of all groups were 2‐month‐old. *p < 0.05, **p < 0.01, ***p < 0.001 in k and l. Data are presented as means ± SEM

**FIGURE 7:**
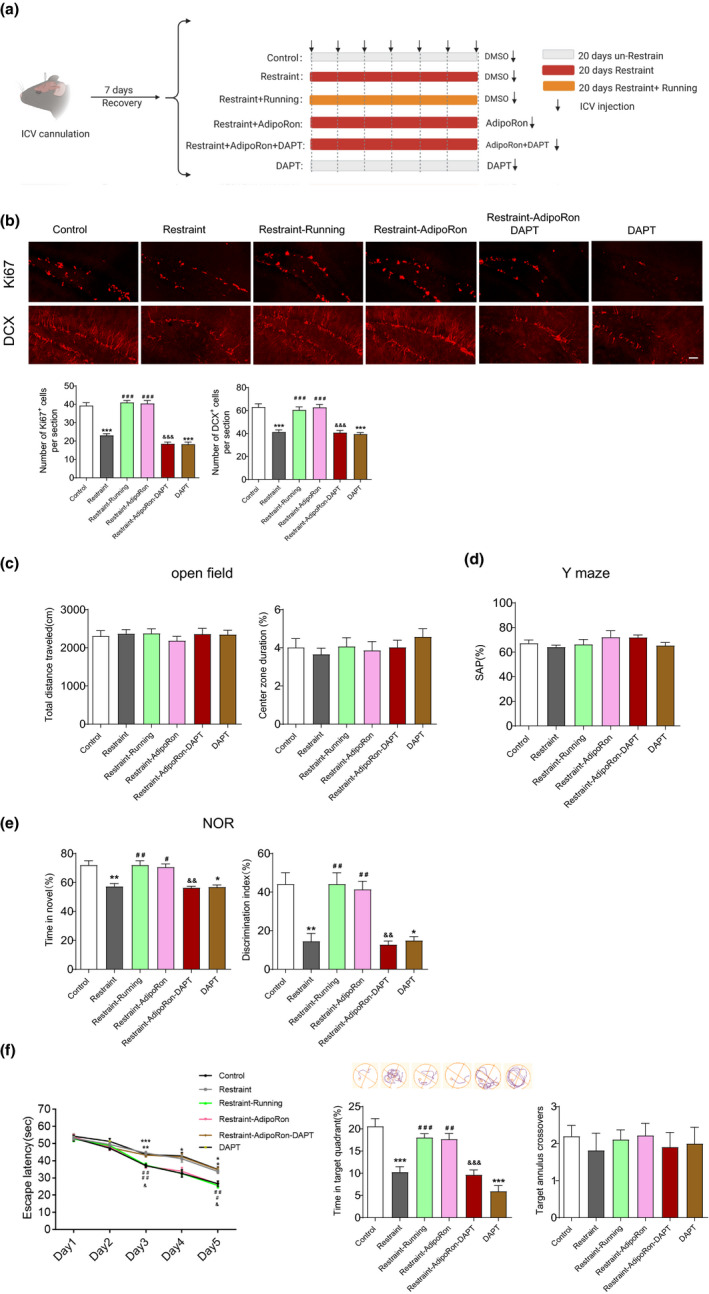
Physical exercise and AdipoRon ameliorate the impairments in hippocampal neurogenesis and cognitive function induced by chronic restraint stress and inhibition of a Notch signaling pathway by DAPT mimics the adverse effects of chronic restraint stress in middle‐aged mice. (a) The experimental protocol and groups used. (b) representative image and quantification of immunohistochemical staining of Ki67 and DCX in DG. Control, *n* = 6; Restraint, *n* = 7; Restraint + Running, *n* = 6; Restraint + AdipoRon, *n* = 6; Restraint + AdipoRon + DAPT, *n* = 7; DAPT, *n* = 6. Mice of all groups were 12‐month‐old. ***p < 0.001 compared with Control; ^###^ *p* < 0.001 compared with Restraint treatment; ^&&&^ *p* < 0.001 compared with Restraint + AdipoRon treatment. (c) Open field test showing the locomotor activity and anxious level after various treatments. Left, total distance travelled in 10 min; Right, percentage of time spent in the center zone. (d) Y‐maze test showing the alternation rate after various treatments. (e) Novel objective recognition test showing the exploring time on new object after various treatments. Control, *n* = 10; Restraint, *n* = 11; Restraint + Running, *n* = 9; Restraint + AdipoRon, *n* = 9; Restraint + AdipoRon + DAPT, *n* = 10; DAPT, *n* = 9 in c, d, and e. Mice of all groups were 12‐month‐old. *p < 0.05, **p < 0.01 compared with Control; ^#^ *p* < 0.05, ^##^ *p* < 0.01 compared with Restraint treatment; ^&&^ *p* < 0.01 compared with Restraint + AdipoRon treatment in e. (f) Morris water maze test showing the escape latency on training days (left), illustrative examples of travel pathways for each group (upper right) and percentage of time spent in target quadrant (lower middle), the times crossing the platform on testing day (right). Control, *n* = 10; Restraint, *n* = 11; Restraint + Running, *n* = 9; Restraint + AdipoRon, *n* = 9; Restraint + AdipoRon + DAPT, *n* = 11; DAPT, *n* = 9. Mice of all groups were 12‐month‐old. In the left, *p < 0.05, **p < 0.01, ***p < 0.001 for Restraint versus Control and DAPT versus Control; ^#^ *p* < 0.05, ^##^ *p* < 0.01 for Restraint versus Restraint + Running and Restraint versus Restraint + AdipoRon; ^&^ *p* < 0.05 for Restraint + AdipoRon versus Restraint + AdipoRon + DAPT. In the middle, ***p < 0.001 compared with Control; ^##^ *p* < 0.01, ^###^ *p* < 0.001 compared with Restraint; ^&&&^ *p* < 0.001 compared with Restraint + AdipoRon. Data are presented as means ± SEM. Scale bar: b, 50 μm

We thus first determined whether Adiponectin‐Notch pathway was inhibited in mice with chronic stress‐induced depression. In our study, chronic restraint stress‐induced depressive‐like behaviors, as evaluated by the sucrose preference test (SPT; Figure 6b) and forced swim test (FST; Figure 6c), while locomotor activity was not affected (Figure 6d). The Western blot and qPCR results showed that chronic restraint stress decreased the level of adiponectin in serum and the Notch signaling in the hippocampus (Figure 6e,f). Immunofluorescence results showed that chronic restraint stress inhibited hippocampal neurogenesis as determined by Ki67‐ and DCX staining (Figure 7b). ICV injection of AdipoRon, activating Notch signaling, ameliorated the hippocampal neurogenesis dysfunction in mice under chronic restraint stress, while inhibiting Notch signaling by DAPT mimicked the detrimental effects of chronic stress on hippocampal neurogenesis (Figure 7b). Taken together, these results indicated that the inhibited Adiponectin‐Notch pathway may be responsible for the decreased hippocampal neurogenesis in chronic stress‐induced depression. Studies have shown that impaired hippocampal neurogenesis is associated with neurodegenerative diseases, and may contribute to cognitive dysfunction (Clelland et al., 2009; Ma et al., 2017). Hence, we next examined whether chronic restraint stress induces cognitive impairment, as evaluated by the Y‐maze, NOR, and Morris water maze tests. The results of the Y‐maze test showed that chronic restraint stress did not affect the total percentage of correct spontaneous alterations (Figure 7d). In the NOR test, the chronic restraint stress group spent less time on novel object investigation than the control group (Figure 7e). In the Morris water maze test, the escape latency of mice in the chronic restraint stress group was significantly increased compared with that of mice in the control group on days 4–5 (Figure 7f, left), and mice in the chronic restraint stress group spent less time in the target quadrant than mice in the control group (Figure 7f, lower middle). The number of target annulus crossovers did not differ significantly (Figure 7f, right). Moreover, ICV injection of AdipoRon improved cognitive function impaired by chronic restraint stress, while DAPT treatment‐induced cognitive dysfunction (Figure 7e,f). These results showed that chronic restraint stress induced both depression and cognitive impairment, and indicated that decreased activity of Adiponectin‐Notch pathway may contribute to the cognitive impairment associated with depression. Collectively, our data supports the hypothesis that chronic stress inhibits Adiponectin‐Notch pathway, which induces impairment of hippocampal neurogenesis and subsequent cognitive dysfunction.

We subsequently explored whether physical exercise can reverse the inhibited Adiponectin‐Notch pathway, decreased hippocampal neurogenesis, and impaired cognitive function in mice under chronic restraint stress. Consistent with previous reports (Kwok et al., 2019), running ameliorated chronic restraint stress‐induced depressive‐like behaviors, as evaluated by the SPT (Figure 6h) and FST (Figure 6i). The Western blot and qPCR results showed that running reversed the chronic restraint stress‐induced decreases in the serum level of adiponectin and the Notch signaling in the hippocampus (Figure 6k,l). Running reversed the decrease in hippocampal neurogenesis (Figure 7b) and the impairment of cognitive dysfunction (Figure 7e,f). Taken together, these results suggest that physical exercise may exert a therapeutic effect on chronic stress‐induced impairment of hippocampal neurogenesis and cognitive function through upregulating Adiponectin‐Notch signaling in the hippocampus.

### 2.9 Adiponectin regulates the expression of ADAM10 and Notch1 through PPARα and JNK pathways respectively

**FIGURE 8:**
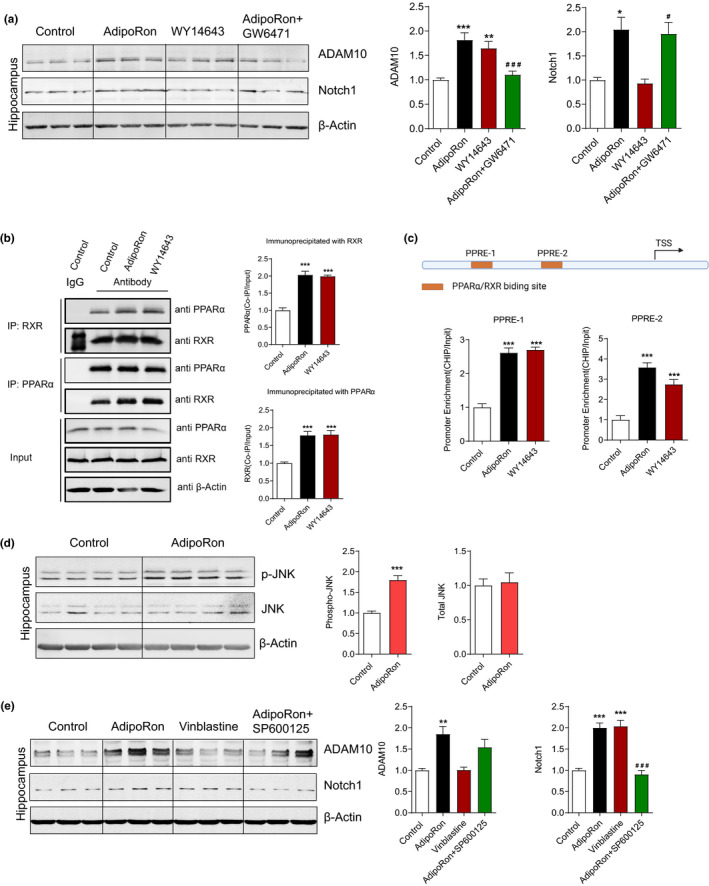
Adipopnectin regulates the expression of ADAM10 and Notch1 through PPARα and JNK pathway respectively. (a) Representative immunoblots and quantification of hippocampal protein levels after chronic ICV injection of AdipoRon, WY14643 (PPARα agonist) and GW6471 (PPARα antagonist) in 12‐month‐old mice, including Notch1 and ADAM10. Control, *n* = 6; AdipoRon, *n* = 6; WY14643, *n* = 7; AdipoRon + GW6471, *n* = 7. *p < 0.05, **p < 0.01, ***p < 0.001 compared with Control; ^#^ *p* < 0.05, ^###^ *p* < 0.001 compared with AdipoRon treatment. (b) Co‐immunoprecipitation results showing chronic ICV injection of AdipoRon or WY14643 enhances the interaction between PPARα and RXR. Control, *n* = 6; AdipoRon, *n* = 6; WY14643, *n* = 7. ***p < 0.001 compared with Control. (c) qPCR results showing chronic ICV injection of AdipoRon or WY14643 Induces the recruitment of RXR to the ADAM10 Promoter. Control, *n* = 6; AdipoRon, *n* = 7; WY14643, *n* = 7. ***p < 0.001 compared with Control. (d) Chronic ICV injection of AdipoRon upregulates the activity of JNK. Control, *n* = 6; AdipoRon, *n* = 6. ***p < 0.001. (e) Representative immunoblots and quantification of hippocampal protein levels after chronic ICV injection of AdipoRon, Vinblastine (JNK agonist) and SP600125 (JNK antagonist) in 12‐month‐old mice, including Notch1 and ADAM10. Control, *n* = 6; AdipoRon, *n* = 7; Vinblastine, *n* = 7; AdipoRon + SP600125, *n* = 7. **p < 0.01, ***p < 0.001 compared with Control; ^###^ *p* < 0.001 compared with AdipoRon treatment. Data are presented as means ± SEM

We next explored the molecular mechanisms by which adiponectin upregulated the expression of ADAM10 and Notch1. A previous study suggests the involvement of peroxisome proliferator‐activated receptor alpha (PPARα), in regulating the expression of ADAM10 (Corbett et al., 2015) whose activity would be increased when adiponectin binding its receptors. We thus first determined whether PPARα mediated the effect of adiponectin on the expression of ADAM10 in middle‐aged mice. Chronic ICV injection (7 times, every 3 days) of WY14643 (PPARα agonist, 1 µl/200 µM) increased the expression of ADAM10, while GW6471 (PPARα antagonist, 1 µl/48 µM) blocked the increased expression of ADAM10 induced by AdipoRon (Figure 8a), suggesting PPARα mediating the effect of AdipoRon on the expression of ADAM10. Neither activating nor inhibiting the PPARα influenced the expression of Notch1 (Figure 8a), suggesting that PPARα may not be involved in the expression regulation of Notch1 by adiponectin. Retinoid x receptor (RXR), the PPARα allosteric heterodimer partner, is also suggested involved in the expression regulation of ADAM10 (Corbett et al., 2015; Tippmann et al., 2009). Co‐IP results showed that chronic ICV injection AdipoRon or WY14643 enhanced the interaction between PPARα and RXR (Figure 8b), and CHIP results showed that chronic ICV injection AdipoRon or WY14643 augmented the recruitment of RXR to the predicted PPAR response element (PPRE) in the ADAM10 promoter as described in a previous report (Corbett et al., 2015; Figure 8c), indicating adiponectin may activate the transcription of ADAM10 through augmented the recruitment of PPARα/RXR heterodimer to the ADAM10 promoter.

Our Western blot results showed that chronic ICV injection (7 times, every 3 days) of AdipoRon in middle‐aged mice increased the activity of c‐Jun N‐terminal kinase (JNK) which is shown involved in the upregulation of Notch1 expression (Xie et al., 2017; Figure 8d). Chronic ICV injection of Vinblastine (7 times, every 3 days; JNK agonist, 1 µl/6 µM) increased the expression of Notch1, while SP600125 (JNK antagonist, 1 µl/8 µM) blocked the increased expression of Notch1 induced by AdipoRon (Figure 8e), suggesting JNK mediating the effect of AdipoRon on the expression of Notch1. Neither activating nor inhibiting the JNK influenced the expression of ADAM10 (Figure 8e), suggesting that JNK may not be involved in the expression regulation of ADAM10 by adiponectin. Collectively, our data suggested that adiponectin may regulate the expression of ADAM10 and Notch1 through PPARα and JNK respectively.

## 3 DISCUSSION

Dementia and depression, both common disorders in the elderly, impact the quality of life for patients and relatives and involve substantial health‐care service and social benefit costs (Barnes et al., 2006; Leonard, 2007). The relationship between depression and dementia is complex with depression having been reported to be both a risk factor and a prodrome for Alzheimer's disease and other dementia's, and also be a common complication of dementia at all stages (Bennett & Thomas, 2014; Panza et al., 2010). The mechanisms of comorbidity of these diseases are still unclear. Research drawing more confident conclusions about the underlying neurobiologic pathways, may pave the way for more effective treatments of both depression and dementia. In this study, we found that adiponectin deficiency is associated with decreased hippocampal neurogenesis and cognitive impairment in both middle‐aged APN‐KO mice and middle‐aged depression mice models. Previous studies showed that adiponectin deficiency in middle‐aged mice leads to learning and memory impairments (Bloemer et al., 2019; Ng et al., 2016), while we revealed that Adiponectin level was correlated with both depressive behaviors and cognitive function in middle‐aged depression mice model induced by chronic stress, suggesting Adiponectin was a potential candidate responsible for both cognitive impairment and depression in elderly. Furthermore, our data suggested that adiponectin was also required for physical exercise‐induced improvements in cognitive function.

Impairment in hippocampal neurogenesis is linked to cognitive dysfunction in both major depressive disorder (MDD) and Alzheimer's disease (AD; Berger et al., 2020; Clelland et al., 2009). Previous studies have suggested the indispensable role of hippocampal neurogenesis in hippocampus‐dependent learning and memory learning (Thuret et al., 2009). A previous report shows that adiponectin knockout doesn't influence basal hippocampal neurogenesis (Yau et al., 2014). However, there is also other study that shows adiponectin deficiency reduces hippocampal neurogenesis, which may be due to the difference in the APN‐KO mice lines (Zhang et al., 2016). Unlike these two studies which were conducted on APN‐KO mice at the age of 8–12 weeks, we performed our experiments on 12‐month‐old APN‐KO mice based on reports that middle‐aged APN‐KO mice, but not young, display hippocampus‐dependent memory and learning impairments (Bloemer et al., 2019; Ng et al., 2016). In our system, we observed a clear relationship among plasma adiponectin level, hippocampal neurogenesis, and cognitive function in middle‐aged APN‐KO mice, middle‐aged mice model of depression, and aged mice (Figures 1, 3, and 7). These observations suggest that impaired hippocampal neurogenesis regulated by adiponectin may be the pathogenesis of cognitive dysfunction associated with depression.

Adiponectin, an adipose‐specific cytokine, could cross the blood‐brain barrier (BBB) from the blood into cerebrospinal fluid (Neumeier et al., 2007). Plenty of studies have demonstrated the beneficial effects of adiponectin on adult neurogenesis (Nicolas et al., 2015; Yau et al., 2018; Zhang et al., 2016) and cognitive function (De Franciscis et al., 2017; Rizzo et al., 2020). However, the mechanism underlying those effects of adiponectin has not been fully elucidated. In this study, we uncovered a novel pathway, Adiponectin‐Notch pathway, which mediated the interaction between adipose and brain. Adiponectin could increase the expression of two key molecules in the Notch pathway, ADAM10 and Notch1, which exerted the beneficial effects on hippocampal neurogenesis and cognitive function (Figures 4 and 5). Moreover, Notch signaling decreased in the hippocampus of both middle‐aged APN‐KO mice and aged mice which displayed impaired hippocampal neurogenesis and cognitive dysfunction (Figures 1, 2, 3), and inhibition Notch signaling by DAPT blocked the beneficial effects of AdipoRon on hippocampal neurogenesis and cognitive function (Figures 5 and 7). Collectively, our results suggest that Notch signaling mediates the effect of adiponectin on hippocampal neurogenesis and cognitive function.

Physical exercise is considered an effective therapeutic alternative to improve cognition in patients suffering from MDD (Olson et al., 2017) or AD (Jia et al., 2019). We expected to find whether Adiponectin‐Notch not only is involved in pathophysiology of cognitive impairment associated with depression, but also contributes to the therapeutic effect of physical exercise on cognitive dysfunction. In our study, physical exercise enhanced Adiponectin‐Notch pathway (Figure 2b,c), increased hippocampal neurogenesis (Figures 2A and 5B), and improved learning and memory ability (Figures 1i,j and 5e,f), showing a correlation between activation of Adiponectin‐Notch pathway and improved cognition by physical exercise. These results were consistent with the previous report (Yau et al., 2014). Furthermore, our data showed that physical exercise could reverse the decreased Adiponectin‐Notch signaling induced by chronic restraint stress (Figure 6), and ameliorate the impairments in hippocampal neurogenesis and cognitive function (Figure 7), which indicated that Adiponectin‐Notch pathway may contribute to the therapeutic effect of physical exercise. To isolate the effect of adiponectin on cognitive function from other exercise components, we subjected APN‐KO mice to our physical exercise protocol. Adiponectin deficiency‐induced decreased Notch signaling, impaired hippocampal neurogenesis and cognitive dysfunction, and diminished the beneficial effects of physical exercise (Figures 1, 2, 3). Similarly, ICV injection of DAPT also blocked those improvements of physical exercise (Figure 5). Together, our data suggests the importance of Adiponectin‐Notch pathway in therapeutic effect of physical exercise on cognitive disorders associated with depression.

Furthermore, we revealed the molecular mechanism by which adiponectin activated the Notch signaling in hippocampus. Limited studies give us some indirect clues that PPARα and JNK are involved in the expression regulation of ADAM10 (Corbett et al., 2015) and Notch1 (Xie et al., 2017) respectively. In this study, we demonstrated that Adiponectin upregulated the expression of ADAM10 and Notch1 through PPARα and JNK respectively.

In conclusion, we revealed a novel mechanism that adiponectin increases hippocampal neurogenesis through activating Notch signaling. In addition, our work suggests that the Adiponectin‐Notch pathway may be involved in chronic stress‐induced hippocampal neurogenesis impairment and cognitive dysfunction and mediate the therapeutic effect of physical exercise.

## 4 MATERIALS AND METHODS

### 4.1 Mice

C57BL/6J mice were purchased from Pengyue lab (Jinan, China), and adiponectin knockout mice were purchased from Gempharmatech Company. The protocols of animal studies were approved by the Institutional Animal Care and Use Committee of Binzhou Medical University Hospital, and performed in compliance with the National Institutes of Health Guide for the Care and Use of Laboratory Animals. Efforts were made to minimize animal suffering, and the number of animals used.

### 4.2 Drugs and antibody

The following primary antibodies were used: Rabbit anti ADAM10 (1:1000, ab1997, Abcam); Goat anti ADAM17 (1:1000, ab13535, Abcam); Rabbit anti Notch1 (1:1000, ab27526, Abcam); Rabbit anti NICD (1:1000, ab8925, Abcam); Goat anti Adiponectin (1:1000, AF1119, R&D Systems); Rabbit anti β‐Actin (1:1000, 4970, Cell Signaling Technology); Rabbit anti Ki67 (1:1000, MA5‐14520, Invitrogen); Rabbit anti Doublecortin (1:1000, ab18723, Abcam); Mouse anti Nestin (1:1000, ab6142, Abcam); Mouse anti NeuN (1:500, MAB377, Millipore); Rabbit anti GFAP (1:1000, ab7260, Abcam); Rabbit anti JNK (1:1000, 9252, Cell Signaling Technology); Mouse anti p‐JNK (1:1000, 9255, Cell Signaling Technology); Rabbit anti PPARα (1:1000, 600‐401‐421, Rockland); Mouse anti RXR (1:1000, sc‐46659, Santa Cruz). The used secondary antibodies used including: Alexa Fluor 546, Goat anti‐Rabbit (1:800, A‐11035, Life Technologies); IRDye^®^ 800CW Donkey anti‐Goat IgG (1:10,000, 925‐32214, LI‐COR Biosciences); IRDye^®^ 800CW Donkey anti‐Rabbit IgG (1:10,000, 925‐32213, LI‐COR Biosciences); IRDye^®^ 680RD Donkey anti‐Mouse IgG (1:10,000, 925‐68072, LI‐COR Biosciences). AdipoRon was purchased from AdipoGen Life Sciences (Cat: AG‐CR1‐0154‐M050) and dissolved in DMSO to a storage solution of 30mM that would be further diluted to the desired final concentration in artificial cerebrospinal fluid (aCSF). N‐[2S‐(3,5‐difluorophenyl) acetyl]‐L‐alanyl‐2‐phenyl‐glycine, 1,1‐dimethylethyl ester (DAPT) was purchased from Cayman chemical company (Cat: 13197) and dissolved in DMSO to a storage solution of 20 mg/ml that would be further diluted to the desired final concentration in artificial cerebrospinal fluid (aCSF). WY14643 was purchased from Abcam (ab141142) and dissolved in DMSO to a storage solution of 100 mM that would be further diluted to the desired final concentration in artificial cerebrospinal fluid (aCSF). GW6471 was purchased from Abcam (ab254317) and dissolved in DMSO to a storage solution of 50 mM that would be further diluted to the desired final concentration in artificial cerebrospinal fluid (aCSF). Vinblastine was purchased from MCE (HY‐13780) and dissolved in DMSO to a storage solution of 50 mg/ml that would be further diluted to the desired final concentration in artificial cerebrospinal fluid (aCSF). SP600125 was purchased from MCE (HY‐12041) and dissolved in DMSO to a storage solution of 50 mM that would be further diluted to the desired final concentration in artificial cerebrospinal fluid (aCSF).

### 4.3 Stereotaxic surgery

For intraventricular cannula implantation, male C57BL/6J mice were anesthetized and mounted onto a stereotaxic frame (KOPF). The skull surface was coated with Kerr phosphoric acid gel etchant (Kerr USA). First, guide cannula was inserted into the lateral ventricle (coordinates: 0.2 mm posterior to the bregma, 1.1 mm lateral to the midline, and 1.6 mm ventral to dorsal). Then, adhesive (GLUMA) was applied onto the skull and cannula surface and Resina fluida (Filtek Z350 XT 3M) was brushed on top with light curing for 45 s using a VALO curing light (Ultradent Products). Finally, dental cement was used to seal cannula and a dummy cannula was inserted into the guide cannula to maintain an unobstructed cannula. After surgery, animals were individually housed and then allowed to recover for 7 days with daily handling. Mice were conscious, unrestrained, and freely moving in their home cages during the microinjections. On an experimental day, a 33‐G stainless‐steel injector connected to a 5‐μl syringe was inserted into the guide cannula and extended 1 mm beyond the tip of the guide cannula. Drugs or vehicle was infused in a volume of 1 μl over 5 min. The injector tips were held in place for an additional 5 min after the end of infusion to avoid backflow through the needle track.

### 4.4 Chronic restraint stress and physical exercise

For the chronic restraint stress procedure, male C57BL/6J experimental mice (12‐month‐old at the start of experiments) were subjected to 2 h of restraint stress each day for twenty consecutive days and individually housed until all experiments had finished. For the physical exercise procedure, male C57BL/6J mice ran 1 h at the speed of 12 m/min each day for twenty consecutive days after a 4‐days adaptation to the treadmill.

### 4.5 qRT‐PCR

Total RNA from the hippocampus was extracted with Tissue RNA Kit (OMEGA, Cat: R6688‐02) and was reverse transcripted into cDNA using RevertAid First Strand cDNA Synthesis Kit (Thermo Scientific™, Cat: K1622). qRT‐PCR was performed using the AceQ qPCR SYBR Green Master Mix (Vazyme Biotech, Cat: Q141‐02) and StepOnePlus™ Real‐Time PCR System (Applied Biosystems). The primer sequences used for qRT‐PCR were as follow: Notch1, forward‐5′‐CAGGAAAGAGGGCATCAG‐3′, reverse‐5′‐AGCGTTAGGCAGAGC

AAG‐3′; Notch2, forward‐5′‐GCAGGAGCAGGAGGTGATAG‐3′, reverse‐5′‐GCGTTTCTTGGACTCTCCAG‐3′; Notch3, forward‐5′‐GTCCAGAGGCCAAGA

GACTG‐3′, reverse‐5′‐CAGAAGGAGGCCAGCATAAG‐3′; Notch4, forward‐5′‐CTCTGCAGCCCTGGCTATAC‐3′, reverse‐5′‐GGCATCGAGCAGTGTGTG‐3′; ADAM10, forward‐5′‐CCTGCCATTTCACTCTGTCATTTA‐3′, reverse‐5′‐GTGC

CCGGGCTCCTTCCTCTACTC‐3′; ADAM17, forward‐5′‐TGAGCGGTGACCAC

GAGAATAATA‐3′, reverse‐5′‐CATAATACCCGGGTCACACTCCTC‐3′; Hes1, forward‐5′‐AATGCCGGGAGCTATCTTTCT‐3′, reverse‐5′‐CCAGCCAGTGTCA

ACACGA‐3′; Hes5, forward‐5′‐AGTCCCAAGGGAGAAAAACCGA‐3′, reverse‐5′‐GCTGTGTTTCAGGTAGCTGAC‐3′; Hey1, forward‐5′‐TGAATCCAGATGA

CCAGCTACTGT‐3′, reverse‐5′‐TACTTTCAGACTCCGATCGCTTAC‐3′; HeyL, forward‐5′‐CAGATGCAAGCCCGGAAGAA‐3′, reverse‐5′‐ACCAGAGGCATG

GAGCATCT‐3′; β‐Actin, forward‐5′‐AGCCATGTACGTAGCCATCC‐3′, reverse‐5′‐TGTGGTGGTGAAGCTGTAGC‐3′.

### 4.6 Western blot assay

Mice were decapitated rapidly, and trunk blood was collected in tubes containing 20 μl of 0.5% ethylenediaminetetraacetic acid disodium and centrifuged at 10,000 *g* for 5 min. The supernatant was collected and stored at −80°C. The hippocampus was dissected, frozen in liquid nitrogen, and stored at −80°C before being processed for western blotting. Images were captured and quantitatively analyzed with Odyssey Sa Quantitative Infrared Imaging System (LI‐COR Biosciences). Adiponectin levels were normalized to IgG in the plasma and the protein levels of the hippocampus were normalized to β‐actin.

### 4.7 Immunostaining and cell counting

Mice were anesthetized and transcardially perfused with cold PBS and 4% paraformaldehyde (PFA) sequentially. Mouse brains were maintained in 4% PFA at 4°C overnight and then dehydrated in 30% sucrose for 2 days. Coronal sections (40 μm) containing the target brain region were obtained using a freezing microtome (Leica, CM1950). The sections were incubated in blocking buffer (10% normal goat serum, 0.3% Triton X‐100) for 1 h at room temperature and then incubated with primary antibodies overnight at 4°C, followed by rinsing in PBS buffer and secondary antibody incubation for 4 h at room temperature. The sections were mounted with Gold antifade reagent (Invitrogen, Thermo Fisher Scientific). Images were captured by the Olympus FV10 confocal system (Olympus). For each brain, 7–8 hippocampal sections (every sixth section, approximately from 1.34 to 3.64 mm posterior to bregma) were selected for Ki67, Nestin, DCX, NeuN or GFAP staining as described above. To visualize and quantify staining positive cells, confocal z‐stack images with a step size of 2 μm were captured with a 20× objective. For Ki67 and Nestin quantification, only the cells located within the subgranular zone were counted. For DCX, NeuN, and GFAP quantification, the cells located within the whole dentate gyrus granule cell layer were counted. The immunopositive cells were counted bilaterally by experimenters who were blind to the genotypes or treatments. For each brain, the number of immunopositive cells per section was calculated by dividing the total number of immunopositive cells into all selected sections by the number of selected sections.

### 4.8 Co‐Immunoprecipitation (Co‐IP)

Mice were decapitated rapidly, Hippocampus was collected and transferred to a 2 ml tube (Hippocampus of two mice/one tube). Then added 1.2 ml ice‐cold IP lysis buffer and homogenized, centrifuged at 10,000 *g* for 5 min. Transferring the supernatant to a 1.5 ml tube and, and adding 50 µl Protein A/G Agarose (sc‐2003, Santa Cruz), then rotating the tube in 4℃ for 1 h. Centrifuged at 3000 *g* for 5 min, transferred the supernatant to a new 1.5 ml tube and add 10 µg antibody or mouse IgG. Then rotated the tube in 4℃ overnight. Adding 100 µl Protein A/G Agarose and rotating at room temperature for 1 h, then centrifuging at 3000 *g* for 5 min and got rid of the supernatant. Washed the precipitate three time with IP wash buffer, then added protein loading buffer and boiled for next western blot.

### 4.9 Chromatin Immunoprecipitation (CHIP)

Mice were decapitated rapidly, Hippocampus was collected and transferred to a 2 ml tube (Hippocampus of two mice/one tube). Then added 1.2 ml ice‐cold PBS containing 1% Formaldehyde and added 75 µl Glycine solution (2 M) 15 min later. Centrifuged and got rid of the supernatant, and washed the precipitate with ice‐cold PBS. Then added 1.2 ml nuclear lysis buffer and homogenized, centrifuged at 10,000 *g* for 5 min. Transferring the supernatant to a 1.5 ml tube and getting DNA fragments using ultrasonication. Transferred 400 µl supernatant to a 5 ml tube, and added 4 ml CHIP dilution buffer. Then performed CHIP with PPARα and RXR antibody. qPCR was performed as described previously (Corbett et al., 2015). Primer: PPRE‐1: forward‐5′‐GAGGCCCCGAGAGAGTTATC‐3′; reverse‐5′‐GCAGATGATGACTTAACAGA

AGG‐3′; PPRE‐2: forward‐5′‐CAGCTTTTGCCTTGGTTCTT‐3′; reverse‐5′‐ATTGGGTTCCTTTTCCATCC‐3′.

### 4.10 Sucrose preference test

This task is used to assess anhedonia in depression which is based on the animal's natural preference for sweets. Before beginning testing, Mice were habituated to the presence of two drinking bottles for one week. On an experimental day, water was deprived for three hours. After lights off during the dark cycle, mice have the free choice of either drinking 1% sucrose solution or water for 2 h. Sucrose and water consumption were determined by measuring the weight changes. Sucrose preference was calculated as the ratio of the mass of sucrose consumed versus the total mass of sucrose and water consumed during the test.

### 4.11 Forced swim test

This task is used for assessing the behavioral despair in depression by measuring the immobility time when mice were immersed in a plexiglas cylinder filled with water. On an experimental day, the plexiglas cylinder (25 cm height × 10 cm diameter) was filled with water at a 15 cm depth (24°C ± 1°C). Each mouse was tested for 6 min and video was recorded by a camera directly above. The latency to immobility at the first 2 min and the duration of immobility during the last 4 min were measured. Immobility was defined as no movements except those that maintain their head above water for respiration.

### 4.12 Tail suspension test

This task is used for assessing the behavioral despair in depression by measuring the immobility time when mice were suspended by their tails. On the experimental day, each mouse was suspended within a three‐walled compartment (50 height × 15 width × 15 cm depth) and video was recorded by a camera for 6 min. The degree of depression was assessed by calculating the duration of immobility during the 6 min.

### 4.13 Light‐Dark test

This test is based on the conflict between innate aversion of light and spontaneous exploratory behavior in the novel environment which could be used to evaluate the anxiogenic‐like activity in mice (Bourin & Hascoët, 2003). The apparatus consisted of a polypropylene cage (45 × 27 × 30 cm) and was separated into two compartments, one third for the dark compartment and two thirds for the light compartment. There was an opening between the two compartments (7 × 7 cm). When conducted this test, each mouse was placed in the center of the dark compartment facing away from the opening and video was recorded by a camera for 5 min. The time spent in the light compartment and the number of entries into the light compartment were recorded.

### 4.14 Elevated plus maze

This test was used to measure the anxiety‐like behavior in mice which was based on the natural aversion of open and elevated areas. The elevated plus‐maze comprised two open arms (25 × 5 × 0.5 cm) and two closed arms (25 × 5 × 16 cm), and was elevated 70 cm height from the floor. When conducted this test, each mouse was placed in the center and allowed to freely explore for 5 min. The percentage of time spent in the open arms and the percentage of entries into the open arms were recorded.

### 4.15 Locomotor

This task is used to assess locomotor activity which was performed in SuperFlex open field cages (40 × 40 × 30 cm, Omnitech Electronics Inc.), and mice were allowed 30 min free exploration under illuminated conditions. The total distance traveled was quantified using Fusion version 6.5M software (Omnitech Electronics Inc.).

### 4.16 Open field

This test was performed in an arena (60 × 60 × 40 cm) with even illumination. Mice were allowed free movement for 10 min that was recorded by a camera. The distance traveled in the central zone and the total distance traveled in the arena were analysed using Any‐maze software (Stoelting). The arena was divided into nine squares (3 × 3 grid), and the central square was defined as the central zone.

### 4.17 Novel object recognition (NOR)

Novel object recognition test was used to assess short‐term spatial memory of mice and performed with a slightly modified protocol as described previously (Antunes & Biala, 2012; Liu et al., 2020). Mice received 2 days of habituation in a 45 × 45 cm square arena, and on the third day, they were allowed to explore two identical objects for 10 min (training trial). After 2 hr, one object was replaced by a novel one and the mice were allowed to explore for another 10 min (testing trial). The time spent on each object was then calculated as a percentage of total object exploration.

### 4.18 Y maze

The Y‐maze test was performed with a slightly modified protocol as previously described (Chiba et al., 2009). The apparatus for Y maze was a symmetrical Y Maze (3 arms, 40 × 9 cm with 12 cm‐high walls). The three arms were connected at an angle of 120°. Mice were individually placed at the end of an arm and allowed to explore the maze freely for 10 min. The total arm entries and spontaneous alternation percentage (SAP) were measured. Overlapping triplets of 3‐arm visits were counted as one ‘successful choice’. SAP was defined as a ratio of the number of ‘successful choice’ to the number of total choices (total entry minus two).

### 4.19 Morris water maze (MWM)

The Morris water maze test was performed as previously described (Barnhart et al., 2015; Vorhees & Williams, 2006). The water maze of 150 cm in diameter and 50 cm in height was filled with water (25 ± 0.5°C) to maintained the water surface 1.00 cm higher than the platform (10 cm in diameter). Water was dyed white and the tank was divided into four quadrants and the platform was placed at the center of the designated quadrant. In the acquisition phase (4 trials/day for 5 consecutive days), mice were put into the water from four points in random order every day until they found the platform and stayed for 10 s within 1 min. If the mice cannot find the platform within 1 min, they were guided to the platform. During the retention phase, the platform was removed from the pool, and the mice were placed in water from the opposite quadrant of the platform and tested for 1 min. Videos were recorded and analysed by Any‐maze software (Stoelting).

### 4.20 Statistical analyses

Statistical analysis was performed with graphpad prism software. Results are presented as mean ± standard error of mean (SEM). Shapiro–Wilk test and *F* test were used to test the normality and equal variance assumptions, respectively. For normally distributed data, two‐tailed *t*‐tests were used to assess differences between two experimental groups with equal variance. For a two‐sample comparison of means with unequal variances, two‐tailed *t*‐tests with Welch's correction were used. One‐way analyses of variance (ANOVAs) followed by Tukey's multiple comparisons test were used for analysis of three or more groups. For non‐normally distributed data, Mann–Whitney *U*‐tests were performed to compare two groups. For analysis of three or more groups with non‐normally distribution, the Kruskal–Wallis test followed by Dunn's multiple comparisons test was used. For multiple groups, two way or two‐way repeated‐measures ANOVAs followed by Tukey's multiple comparisons test were used. *p* < 0.05 was considered statistically significant.

## CONFLICT OF INTEREST

None declared.

## AUTHOR CONTRIBUTIONS

B.L., H.Y. and J.C. conceived this study and designed the experiments. B.L., H.Y. and J.C. wrote the manuscript. J.Y., L.S., J.W., F.S., W.W., D.W., X.F., D.L., Z.X., and C.Q. performed the experiments and analyzed the data.

## Data Availability

The data that support the findings of this study are available from the corresponding author upon reasonable request.
